# Receptor-like Kinase GOM1 Regulates Glume-Opening in Rice

**DOI:** 10.3390/plants14010005

**Published:** 2024-12-24

**Authors:** Xinhui Zhao, Mengyi Wei, Qianying Tang, Lei Tang, Jun Fu, Kai Wang, Yanbiao Zhou, Yuanzhu Yang

**Affiliations:** 1 College of Agronomy, Hunan Agricultural University, Changsha 410128, China; zhaoxinhui84@126.com; 2 Key Laboratory of Southern Rice Innovation & Improvement, Ministry of Agriculture and Rural Affairs/Hunan Engineering Laboratory of Disease and Pest Resistant Rice Breeding, Yuan Longping High-Tech Agriculture Co., Ltd., Changsha 410001, China; 3 College of Life Sciences, Hunan Normal University, Changsha 410081, China; 4 College of Agriculture, Nanjing Agricultural University, Nanjing 210095, China; 5 State Key Laboratory of Hybrid Rice, Hunan Hybrid Rice Research Center, Changsha 410125, China; 6 Shanghai Collaborative Innovation Center of Agri-Seeds, School of Agriculture and Biology, Shanghai Jiao Tong University, Shanghai 200240, China

**Keywords:** glum-opening, lodicule, anthesis, jasmonic acid, carbohydrate, rice

## Abstract

Glume-opening of thermosensitive genic male sterile (TGMS) rice (*Oryza sativa* L.) lines after anthesis is a serious problem that significantly reduces the yield and quality of hybrid seeds. However, the molecular mechanisms regulating the opening and closing of rice glumes remain largely unclear. In this study, we report the isolation and functional characterization of a glum-opening mutant after anthesis, named *gom1*. *gom1* exhibits dysfunctional lodicules that lead to open glumes following anthesis. Map-based cloning and subsequent complementation tests confirmed that GOM1 encodes a receptor-like kinase (RLK). *GOM1* was expressed in nearly all floral tissues, with the highest expression in the lodicule. Loss-of-function of *GOM1* resulted in a decrease in the expression of genes related to JA biosynthesis, JA signaling, and sugar transport. Compared with LK638S, the JA content in the *gom1* mutant was significantly reduced, while the soluble sugar, sucrose, glucose, and fructose contents were significantly increased in lodicules after anthesis. Together, we speculated that GOM1 regulates carbohydrate transport in lodicules during anthesis through JA and JA signaling, maintaining a higher osmolality in lodicules after anthesis, which leads to glum-opening.

## 1. Introduction

Rice (*Oryza sativa* L.) serves as a critical staple food for over half of the global population. Hybrid rice, with a 20–30% higher yield compared to conventional rice, represents a significant milestone in agricultural development and has played a crucial role in enhancing global food security [1,2]. Rice heterosis utilization can be achieved through two systems: a three-line system and a two-line system. Compared to traditional three-line hybrid rice, the two-line hybrid rice has garnered increasing attention due to its independence from restorer genes and its greater efficiency in producing hybrid seeds [3]. The development of a two-line system using photoperiod- and thermosensitive genic male-sterile lines (PGMS/TGMS) expands the application of heterosis both within and between rice subspecies. The two-line hybrid rice achieves a 5–10% increase in yield compared to the three-line system [4]. Longke638S (LK638S) is an indica TGMS line developed in the 2000s. It is characterized by a low critical sterility-inducing temperature (below 23.5 °C), a high outcrossing rate, and excellent combining ability [5]. However, during the heading and flowering periods, LK638S exhibits poor glume closure when exposed to high-temperature and high-humidity conditions, resulting in a decrease in seed thousand-grain weight, susceptibility to insect and pathogen infections, and easy sprouting, thereby reducing the yield and quality of hybrid rice seed production.

The palea and lemma are distinctive organs in grass plants that interlock to protect the inner floret and kernel from attack by pathogens or insects and environmental stresses until harvest [6]. However, pollination requires that the lemma and palea be separated for 1–1.5 h/day during anthesis. This occurs as a result of a temporary increase in the turgidity of the lodicule [7,8,9]. The lodicule is a critical organ that controls the opening and closing of rice spikelets [10,11]. The glume opens as the lodicules swell upon water absorption. After anthesis, the glume closes as the lodicules lose water and other content, leading to withering. The lodicule is composed of numerous parenchymal cells and some vascular bundles. Parenchyma cells are interconnected via plasmodesmata, which are crucial for the rapid swelling and transport of substances throughout the lodicule. The increase in soluble sugars in lodicule cells results in increased cellular osmotic pressure, which may serve as a critical driving force for water absorption by the lodicule [10]. However, how lodicule cells lose or swell their water to regulate the opening and closing of the glume has remained unexplored.

Jasmonic acid (JA) is a lipid-derived hormone involved in plant defense responses and various developmental processes, including root growth, seed size, stamen, and floret organ development, as well as adaptation to various biotic and abiotic stresses [12,13,14,15]. Jasmonates (JAs) include JA and its derivatives, such as methyl jasmonate (MeJA), jasmonoyl-L-isoleucine (JA-Ile), and 12-hydroxy jasmonoyl sulfate (12-HSO4-JA). JA is derived from linolenic acid, which is released from galactolipids by phospholipase A1 (DAD1) on the chloroplast membranes and then converted to OPDA (12-oxo-phytodienoic acid) through a series of enzymatic reactions catalyzed by lipoxygenase (LOX), allene oxide cyclase (AOC), and allene oxide synthase (AOS) [16]. Subsequently, OPDA is further converted to JA through 12-oxophytodienoic acid reductase (OPR) and three β-oxidation reactions within peroxisomes [12,17]. Finally, JAs are transported to the cytoplasm, where they undergo further metabolism to generate various derivatives. Numerous studies in plants have established a framework for JA perception and signal transduction, in which the JA receptor CORONATINE INSENSITIVE 1 (COI1), an F-box protein, perceives JA and promotes the formation of COI1-JAZ (JASMONATE ZIM-DOMAIN) protein complexes, leading to degradation of JAZ proteins and release of a set of transcription factors, including the MYC family, which subsequently activate the expression of downstream JA responsive genes [18,19,20].

Plant receptor-like protein kinases (RLKs) constitute one of the largest and most diverse superfamilies of plant proteins, with over 610 members in Arabidopsis and over 1131 in rice [21]. RLKs consist of an extracellular N-terminal domain that functions as a ligand-binding site, a single membrane-spanning region, and a conserved C-terminal cytoplasmic protein kinase domain [22]. RLKs are involved in the pathogen defense response, adaptation to abiotic stresses, hormone perception, developmental regulation, prevention of self-pollination, and quantitative yield components [23,24,25,26,27,28]. Based on the structural characteristics of the extracellular domain, RLKs are classified into 44 subfamilies, including the self-compatibility domain (S-domain), leucine-rich repeat (LRR), lectin-like, and wall-associated kinase (WAK) [29,30]. Although RLKs play crucial roles in cellular processes in plants, most of their features remain unclear.

Here, we isolated a rice mutant, *gom1*, that exhibits glume-opening after anthesis, associated with a delayed cell degradation process in the lodicules. Map-based cloning showed that GOM1 encodes a typical LRR-RLK that is essential for regulating the opening and closing of rice glume during anthesis. Our results demonstrated that GOM1 regulated carbohydrate transport in lodicules during anthesis through JA and JA signaling, maintaining a higher osmolality in lodicules after anthesis, which leads to glum-opening.

## 2. Results

### 2.1. Characterization of the gom1 Mutant

**Figure 1:**
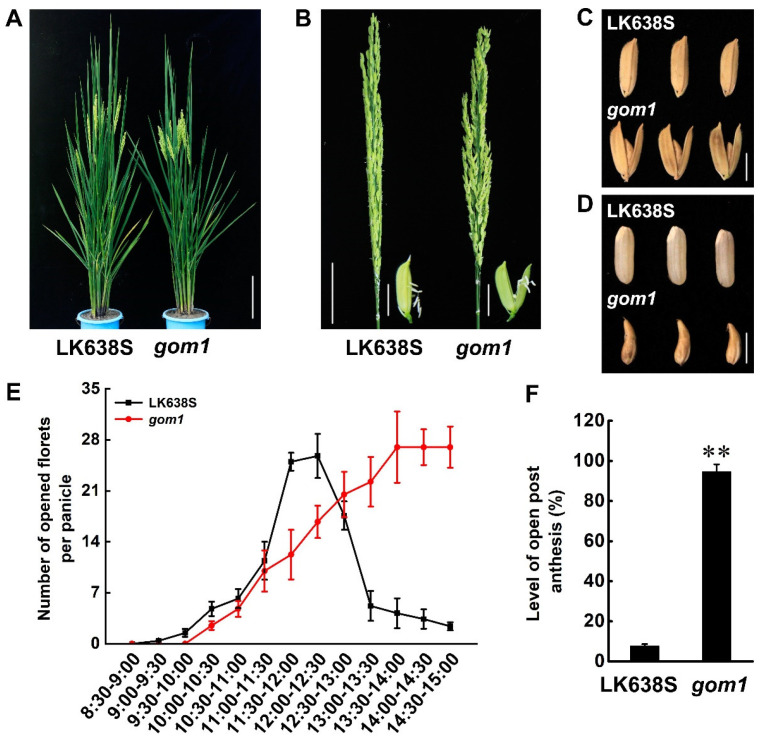
Phenotypic comparison of the LK638S and *gom1* mutant. (**A**) Plant architecture of LK638S and *gom1* mutant plants. Scale bars, 20 cm. (**B**) Comparison of LK638S and *gom1* mutant panicles post-anthesis. Scale bars: 5 cm for panicles and 5 mm for glumes. (**C**,**D**) The *gom1* mutant set up malformed seeds within open glumes compared with LK638S. Scale bar, 5 mm. (**E**) Comparison of flowering habits between the LK638S and *gom1* mutant (*n* = 6). (**F**) Comparison of glume-opening in LK638S and *gom1* mutant following anthesis. Data are presented as mean ± SD (*n* = 3, ** *p* ≤ 0.01, Student’s *t*-test).

**Figure 2:**
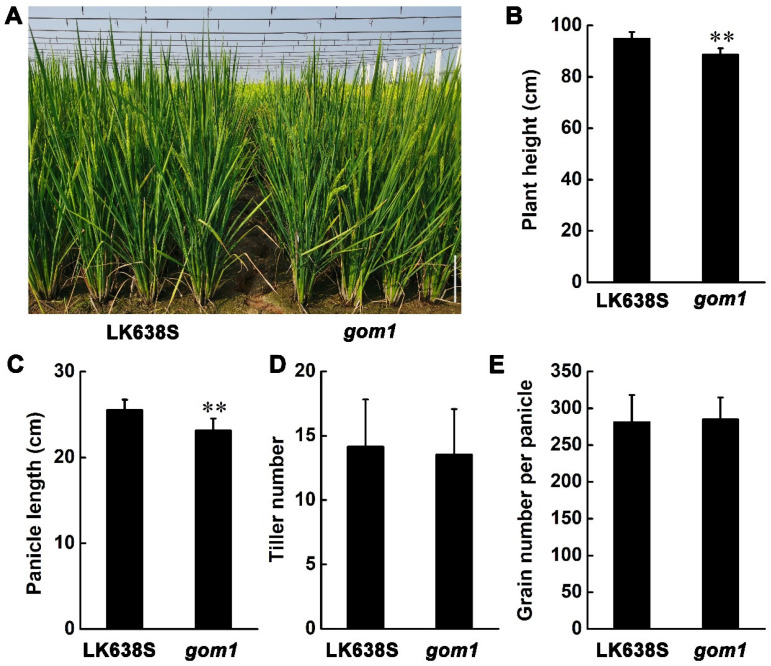
Comparison of major agronomic traits between LK638S and the gom1 mutant in Changsha. (**A**) Phenotypes of LK638S and *gom1* mutant at the heading stage. Scale bar, 20 cm. (**B**–**E**) The major traits, including plant height (**B**), panicle length (**C**), tiller number (**D**), and grain number per panicle (**E**), are shown in histograms. In 2023, agronomic traits were examined in a paddy field located in Guanshan village (28°19′32″ N, 112°40′38″ E), Changsha. Data are presented as the mean ± SD (*n* = 20). ** indicates a significant difference (*p* < 0.01 from Student’s *t*-test).

By screening a ^60^Co γ-ray-mutagenized M_2_ library against the background of Longke638S (LK638S), an *indica* thermosensitive genic male sterile (TGMS), we identified a mutant with dysfunctional lodicules that led to open glumes following anthesis (Figure 1A,B) and named it *gom1* (*glum-open mutant 1*). Due to the opening of the glumes in the *gom1* mutant, the seed was deformed (Figure 1C,D). Comparing the flowering habits of the LK638S and *gom1* mutant, it was found that the glumes of LK638 and *gom1* mutant initiate opened at 9:00 and 10:00, respectively (Figure 1E). The diurnal flower opening time (DFOT) of *gom1* mutant was 1.0 h later than that of LK638S. Moreover, after 1:30 p.m., the glumes of LK638S were mostly closed, while the *gom1* mutant was unable to close normally. The glumes open rate of LK638S is only 7.64%, while the glumes closure rate of the *gom1* mutant is 94.62% following anthesis (Figure 1F). We further investigated whether the *gom1* mutant could affect rice growth. We investigated the agronomic traits of the *gom1* mutant under natural high-temperature conditions in Changsha and natural low-temperature conditions in Lingshui. In Changsha, compared with LK638S, the plant height and panicle length in the *gom1* mutant were significantly lower (Figure 2A–C). However, there was no significant difference in the tiller number and grain number between LK638S and the *gom1* mutant (Figure 2D,E). In Lingshui, compared with LK638S, the plant height, panicle length, seed setting rate, and grain yield were significantly lower in the *gom1* mutant, and tiller number and grain number per panicle were significantly higher in the gom1 mutant (Figure S1). These results indicated that the *gom1* mutant not only fails to properly close the glume following anthesis but also affects growth.

### 2.2. The Lodicules of gom1 Mutant Delayed Withering After Anthesis

**Figure 3:**
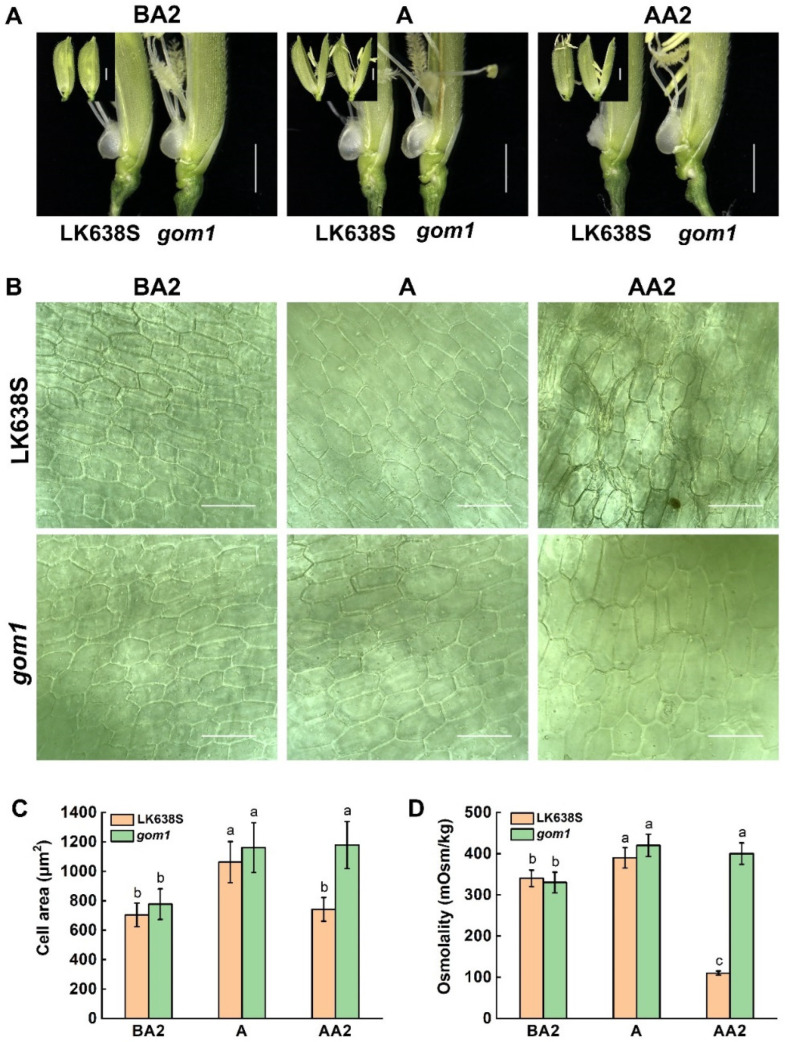
The dynamic process of the glumes and lodicules in LK638S and *gom1* plants. (**A**) The dynamic process of the opening of the glumes and the size of the lodicules in LK638S and *gom1* plants at different stages of anthesis. BA2, 2 h before anthesis; A, anthesis; AA2, 2 h after anthesis. Scale bar: 2 mm. (**B**) The dynamic process occurring in the epidermal cells of the lodicules in LK638S and *gom1* plants at different stages of anthesis. Scale bar: 50 μm. (**C**) The average area of the epidemic cells of the lodicules in LK638S and *gom1* plants at different stages of anthesis. (**D**) Osmolality measurements of lodicules in LK638S and *gom1* plants at different stages of anthesis. Data are presented as the mean ± SD (*n* = 10). Lowercase letters indicate significant differences at *p* < 0.05. Statistical significance was determined using the Student’s *t*-test.

Previous studies have shown that the lodicule is an important organ that determines the opening and closing of rice spikelets during anthesis [31]. To determine morphological alterations in lodicules between the *gom1* mutant and LK638S, we divided the anthesis process into three stages: 2 h before anthesis (BA2), anthesis (A), and 2 h after anthesis (AA2). Overall, the lodicules of both LK638S and *gom1* mutant were in a swollen state at the BA2 and A stages (Figure 3A). The lodicules of LK638S rapidly withered after anthesis; however, the lodicules of the *gom1* mutant remained swollen at AA2 stages (Figure 3A). We further observed cytological changes in lodicules at different time points using microscopy. As with the changes in lodicule size, the size of the lodicule cells changed over time. The lodicule cells of both LK638S and *gom1* gradually expanded from the BA2 to A stages, and then the lodicule cells of LK638S developed an irregular, disordered appearance at the AA2 stages (Figure 3B,C). In contrast to the LK638S, the lodicule cell size of the *gom1* did not show significant changes from the A stage to the AA2 stage.

Considering the persistent swelling lodicules after the A stage in the *gom1* mutant, the osmolality of lodicules was measured at different anthesis stages, and it was found that the osmolality values in the *gom1* mutant increased gradually from the BA2 to the A stage, after which there was no significant change from the A to AA2 stage (Figure 3D). However, the osmolality values of LK638S showed a maximum level at the A stage, after which there was a sharp decline to the AA2 stage. These results suggested that *GOM1* may affect the swelling of lodicules by regulating its osmolality, thereby affecting the opening and closing of the glume during anthesis in rice.

### 2.3. Map-Based Cloning of the GOM1 Locus

**Figure 4:**
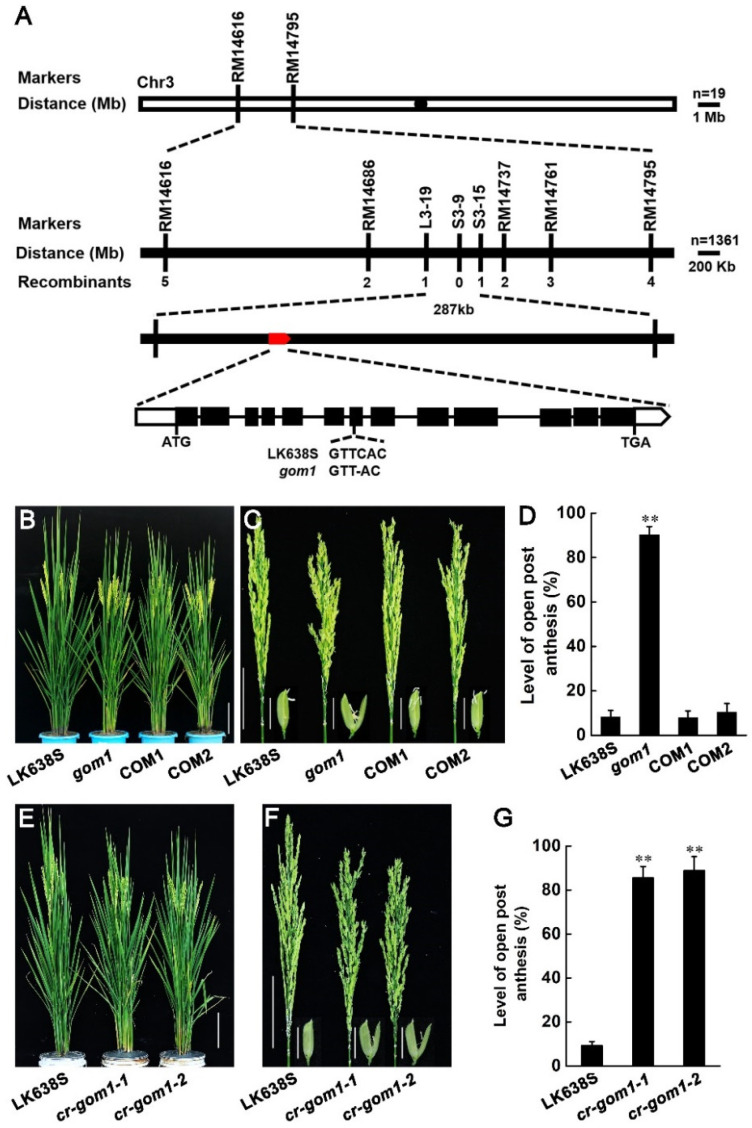
Map-based cloning of *gom1*. (**A**) The *GOM1* locus was mapped to chromosome 3 within a region of 287 kb. A 1 bp deletion in the seven exons led to a premature stop codon. The candidate open reading frame is highlighted in red. (**B**–**D**) Complementation tests rescued the *gom1* phenotypes. Whole-plant morphology (**B**), Panicle and spikelet morphology (**C**), and percentage of glume open following anthesis (**D**). Data are presented as mean ± SD (*n* = 3, ** *p* ≤ 0.01, Student’s *t*-test). (**E**–**G**) Knockout lines of GOM1 showed an open glume phenotype. Whole-plant morphology (**E**), panicle and spikelet morphology (**F**), and percentage of glume open following anthesis (**G**). Data are presented as mean ± SD (*n* = 3, ** *p* ≤ 0.01, Student’s *t*-test).

Crossing of *gom1* with LK638S yielded an F_2_ population in which the segregation ratio of LK638S and the aberrant anthesis phenotype was 3:1 (χ^2^ = 0.67 < χ^2^ _0.05,1_; 0.5 > *p* > 0.1; null hypothesis: fit 3:1). This result suggests that the *gom1* mutant phenotype was due to the recessive mutation of a single locus. To identify the gene responsible for the observed *gom1* mutant phenotype, we developed an F_2_ mapping population by crossing the *gom1* mutant with the TGMS Guangzhan63-4S. The candidate locus was directly mapped to the short arm of chromosome 3 between SSR markers RM14616 and RM14795 (Figure 4A). Further analysis of 1361 F_2_ individuals exhibiting the *gom1* mutant phenotype narrowed down the target gene within a 287 kb region between markers L3 and 19 and S3–15. By comparing the second-generation genome resequencing sequences of LK638S and the *gom1* mutant, we found that there is a 1 bp deletion at the seven exons of LOC_Os03g16010, which encodes a typical LRR-RLK within the candidate interval of the *gom1* mutant, resulting in a frameshift mutation and premature translation termination (Table S2).

To confirm that LOC_Os03g16010 is indeed GOM1, the genomic DNA, including the 2589 bp 5′ flanking sequence, coding region, and 314 bp downstream sequence, was amplified from LK638S and transformed into a *gom1* mutant background. The phenotypes of the obtained transgenic plants were evaluated. Complete rescue of the aberrant anthesis phenotype in the transgenic *gom1* mutants provides compelling evidence that LOC_Os03g16010 is the GOM1 gene responsible for the mutant phenotypes described above (Figure 4B–D). For verification, two independent knockout transgenic lines were generated using the CRISPR/Cas9 system in LK638S (Figures S2 and S3), and the phenotypes were similar to that of *gom1* (Figure 4E–G), confirming that LOC_Os03g16010 is indeed the causal gene of the *gom1* mutant.

### 2.4. The Expression Profiles of GOM1

**Figure 5:**
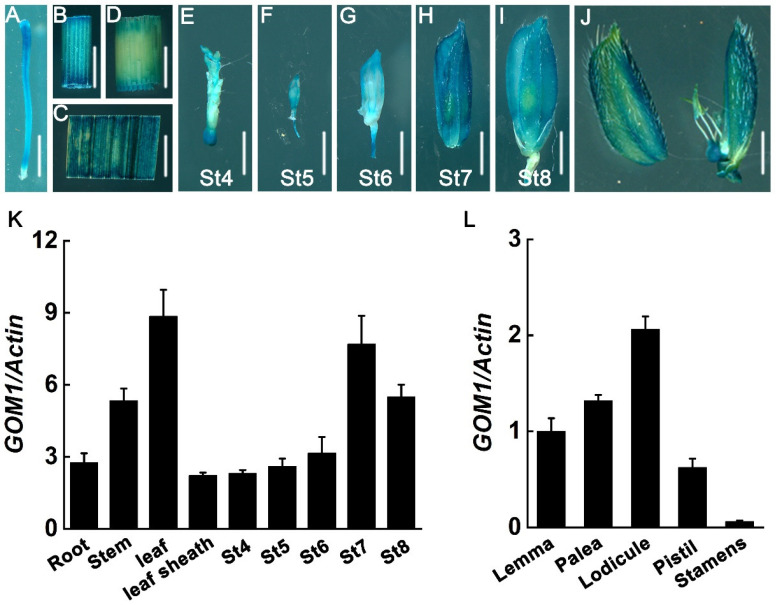
*GOM1* expression pattern in rice. (**A**–**J**) *GOM1* expression revealed by GUS staining in *GOM1* promoter-GUS transgenic plants. Root (**A**), stem (**B**), leaf (**C**), leaf sheath (**D**), and young spikelet at stage 4 to stage 8 (St4 to St8) (**E**–**I**) and a mature spikelet (**J**). Bar = 2 mm; (**K**) The expression level of *GOM1* in various tissues of Kitaake. (**L**) The expression level of *GOM1* in mature spikelet tissues of Kitaake. Data in (**K**–**L**) are presented as mean ± SD (*n* = 3).

To examine the tissue-specific expression pattern of *GOM1*, rice transgenic plants carrying the *pGOM1::GUS* reporter construct were used, in which the 2000 bp *GOM1* promoter region was fused to the *GUS* gene. Histochemical staining revealed that *GOM1* was expressed in the root, stem, leaf, leaf sheath, and early-stage spikelets (stages 4–8), according to the classification of rice anther development [32] (Figure 5A–I), with the highest expression level in the leaf, which was further confirmed by RT-qPCR (Figure 5K). Since GOM1 modulates the opening and closing of the glume by regulating the swelling of lodicules, the expression of *GOM1* in rice floral organs was also investigated. The GOM1-GUS signals in the lemma, palea, lodicule, pistil, and stamens were detected (Figure 5J). The *GOM1* mRNA expression pattern in the rice floral organs was detected using RT-qPCR. *GOM1* was expressed in nearly all floral tissues, with the highest level of expression in the lodicule and the lowest level of expression in the stamens (Figure 5L). The expression of *GOM1* in floral organs supports the important functions of *GOM1* during spikelet development.

### 2.5. Mutation of GOM1 Compromises the Expression of Genes Related to JA Signal Pathway

**Figure 6:**
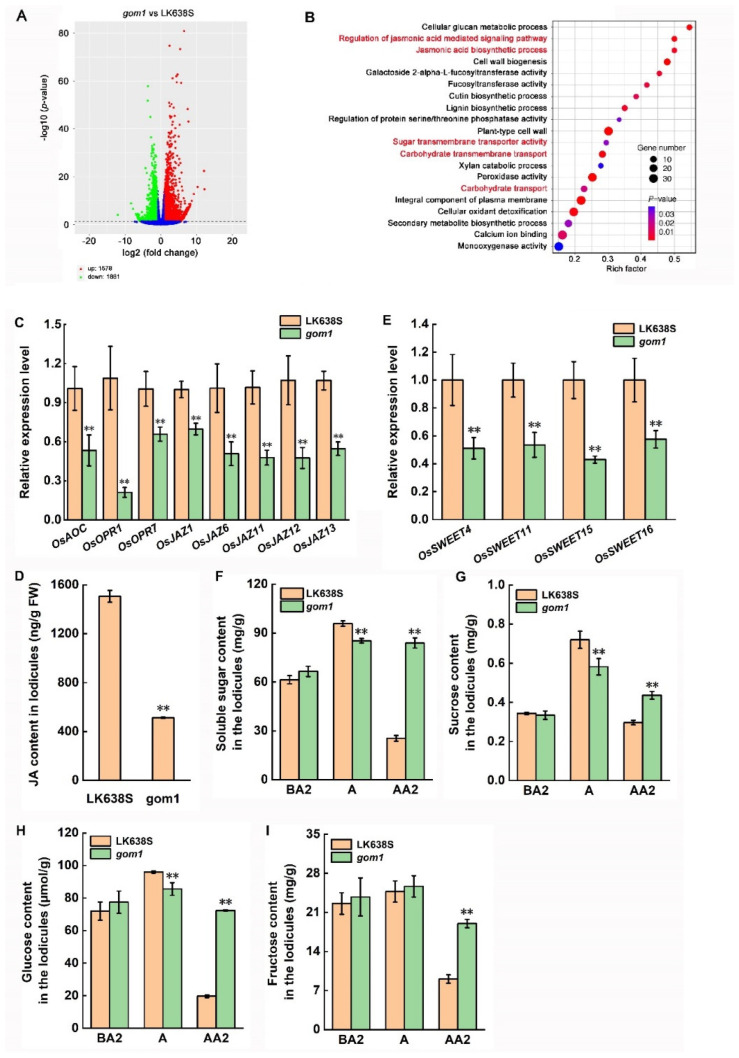
Transcriptomic analyses of lodicules from LK638S and *gom1* mutant. (**A**) Volcano plots comparing the transcriptomes of LK638S with the *gom1* mutant. *X*-axis and *Y*-axis represent log2 fold change (FC) and −log10 (*p*-value), respectively. The green dots represent downregulated DEGs, while the red dots indicate upregulated DEGs. The blue dots indicate no significant difference in transcriptomes. (**B**) GO enrichment analysis of DEGs with a cut-off value of *p* < 0.05. Notably, genes involved in JA biosynthesis and signaling pathways, as well as carbohydrate transport (highlighted in red), were significantly enriched. (**C**) qRT-PCR analysis of the expression levels of JA synthesis and signal pathway genes at the AA2 stage. (**D**) JA content in lodicules of LK638S and *gom1*. (**E**) qRT-PCR analysis of expression levels of *OsSWEET* genes at the AA2 stage. (**F**–**I**) Soluble sugar (**F**), sucrose (**G**), glucose (**H**), and fructose (**I**) levels in lodicules of LK638S and *gom1* at different stages of anthesis. Data in (**C**–**I**) are presented as mean ± SD (*n* = 3, ** *p* ≤ 0.01, Student’s *t*-test).

To understand the mechanisms and downstream components of the *GOM1*-mediated opening of the glume, transcriptome deep sequencing (RNA-seq) analysis was performed using lodicules from LK638S and *the gom1* mutant at the AA2 stage. The criteria for significantly differentially expressed genes (DEGs) were set at a (log2 scale)-fold change (FC) value of >1 or <−1 and an adjusted *p*-value < 0.05. Applying these criteria, we identified 3459 DEGs (1578 upregulated and 1881 downregulated) in the *gom1* mutant compared with LK638S. These DEGs are visualized in a volcano plot, which illustrates the asymmetry between upregulated (red) and downregulated (green) DEGs (Figure 6A). Gene ontology (GO) enrichment analysis revealed multiple significantly enriched pathways (*p* < 0.05), among which the metabolic pathways related to carbohydrates and JA were particularly prominent, including the JA signaling pathway, JA biosynthesis, sugar transmembrane transporter activity, carbohydrate transmembrane transport, and carbohydrate transport (Figure 6B). The JA biosynthetic genes (*OsAOC1*, *OsOPR1*, and *OsOPR7*), JA signaling pathway genes (*OsJAZ1*, *OsJAZ6*, *OsJAZ11*, *OsJAZ12*, and *OsJAZ13*), and *OsSWEET* genes related to carbohydrate transport (*OsSWEET4*, *OsSWEET11*, *OsSWEET5*, and *OsSWEET16*) identified by RNA-seq were further examined for their expression changes in the LK638S and *gom1* mutant at the AA2 stage by RT-qPCR. Compared with LK638S, the expression levels of the JA biosynthetic and JA signaling pathway genes and *OsSWEET* genes were significantly decreased in the *gom1* mutant (Figure 6C,E), which is consistent with the RNA-seq data (Table S3). To confirm whether GOM1 was involved in JA biosynthesis, we compared the endogenous JA content of lodicules in LK638S and *gom1* mutant. JA levels in the *gom1* mutant were significantly lower than those in LK638S, with differences of up to 3-fold (Figure 6D). SWEETs, a novel class of sugar transporters, function as bidirectional uniporters that regulate the import and efflux of sugars across cell membranes [33]. Previous studies have shown that *OsSWEET4* is involved in the transport of glucose and fructose [34], and *OsSWEET11*, *OsSWEET5*, and *OsSWEET16* are involved in the transport of sucrose [35,36]. Thus, the soluble sugars, sucrose, glucose, and fructose contents were measured in the lodicules of LK638S and *gom1* mutant. The contents of soluble sugar, sucrose, glucose, and fructose of both LK638S and *gom1* mutant gradually increased from BA2 to A stages, but the sugar levels of LK638S sharply decreased after anthesis, while in the *gom1* mutant, the contents of sugars were significantly higher than that in LK638s at AA2 stage, which is consistent with the expression of *SWEETs* genes in LK638S and *gom1* mutant at AA2 stage (Figure 6F–I). These results suggested that GOM1 may regulate the opening and closing of glume by modulating the sugar content of the lodicule through the JA biosynthetic and JA signaling pathways.

## 3. Discussion

The lodicule is a crucial organ that controls the opening and closing of rice spikelets during anthesis [10,11]. Spikelet opening in rice is mainly caused by lodicule expansion due to water accumulation, which results from a sudden osmotic pressure increase from starch and soluble sugar buildup in lodicule cells [37]. After anthesis, the glumes can close due to the loss of water, starch, and soluble sugar from the lodicule, which then becomes withered [37]. The lodicule is composed of numerous parenchymal cells and vascular bundles. Parenchyma cells are interconnected via plasmodesmata, which are vital for rapid swelling and substance transport throughout the lodicule [31].

In this study, we identified a rice *gom1* mutant that displayed glume-opening after anthesis (Figure 1A–C). GOM1 encodes a typical LRR-RLK. The lodicules of both LK638S and *gom1* mutants were in a swollen state at the BA2 and A stages (Figure 3A). However, the lodicules of LK638S rapidly withered after anthesis, while the lodicules of the *gom1* mutant remained swollen. In addition, the osmolality values in the *gom1* mutant increased gradually from the BA2 to A stage, after which there was no significant change from the A to AA2 stage (Figure 3D). The osmolality values of LK638S showed a maximum level at the A stage, followed by a sharp decline at the AA2 stage. It is noteworthy that the levels of soluble sugar, sucrose, glucose, and fructose of both LK638S and *gom1* mutant gradually increased from the BA2 to A stages, but the sugar levels of LK638S decreased sharply after anthesis, while in the *gom1* mutant, the amounts of sugars were significantly higher than those in LK638s at the AA2 stage (Figure 6F–I). These results suggested that the lodicules of the *gom1* mutant maintain a high osmotic pressure caused by soluble sugar accumulation after anthesis, leading to the lodicules swelling and preventing the glume-closing.

JA and its derivatives are lipid-derived hormones that play a crucial role in regulating flower development, particularly in the development of anthers [13,33,38,39,40]. The accumulation of osmotic regulation substances, such as potassium ion and other inorganic ion, as well as soluble sugars in lodicules, has been reported to be primarily mediated by JA or JA signaling in lodicules, eventually contributing to lodicule expansion and glume-opening [10,11]. *OsOPR7* encodes a 12-oxophytodienoate reductase, a critical enzyme in the JA biosynthesis pathway that converts OPDA to OPC 8:0. Knockout of the rice *OsOPR7* gene resulted in a defect in JA biosynthesis and displayed open glumes with unwithered lodicules through modulating carbohydrate transport in lodicules by JA after anthesis [33]. OsMYB8 induces the transcription of the JA-Ile synthetase OsJAR1, leading to elevated JAIle accumulation [41]. Knockout of the OsMYB8 in Zhonghua 11 (*Oryza sativa* L. subsp. *japonica*) reduced the contents of fructose, sucrose, and total soluble sugars in lodicules. These results suggested that JA can promote sugar transport in lodicules during anthesis. In our study, JA accumulation of the *gom1* mutant in lodicules was significantly lower than that of in LK638S (Figure 6D), which may result from a decrease in the expression of genes related to JA biosynthesis and signaling in the *gom1* mutant (Figure 6C). Similarly, the contents of soluble sugar, sucrose, glucose, and fructose of the *gom1* mutant in lodicules were significantly higher than those of LK638S (Figure 6F–I), which may result from the reduced expression of genes related to carbohydrate transport after anthesis (Figure 6E), leading to the transmembrane transport of carbohydrates is inhibited and the accumulation of carbohydrates in the lodicules. These results indicated that GOM1 may regulate the opening and closing of the glume by modulating the carbohydrate content of the lodicule through the JA biosynthetic and JA signaling pathways.

Previous studies have shown that JA can effectively facilitate DFOT in rice [42,43], and that DFOT is seriously impaired in several reported JA-deficient mutants [33,39,44]. Overexpressing the JA biosynthesis gene *OPDA REDUCTASE 7* (*OsOPR7*) and knocking out the JA inactivation gene *CHILLING TOLERANCE 1* (*OsHAN1*) in Zhonghua 11 resulted in DFOT stimulation for 1 and 2 h, respectively [42]. Knockout of the JA signal suppressor genes *JASMONATE ZIM-DOMAIN PROTEIN 7* (*OsJAZ7*) and *OsJAZ9* led to DFOT occurring 50-min and 1.5-h earlier, respectively [42]. Overexpressing the JA biosynthesis gene *OsAOS1* promotes DFOT and knocks out *OsAOS1*-impaired DFOT [41]. The rice mutant *osjar1*, which is defective in conjugating various amino acids to JA, fails to respond to MeJA-mediated flower opening in rice [39]. In this study, the JA content in *gom1* mutant lodicules was significantly lower than that in LK638S, with differences of up to 3-fold (Figure 6D), indicating that the loss of function of *GOM1* inhibited the synthesis of JA. Furthermore, compared with the LK638S, the expression levels of genes involved in the JA biosynthetic and JA signaling pathway genes were significantly decreased in the *gom1* mutant (Figure 6C,E), which indicated that GOM1 was involved in JA biosynthesis and signaling. During anthesis, the florets of LK638S and the *gom1* mutant initiate opened at 9:00 and 10:00, respectively (Figure 1E). The DFOT of the *gom1* mutant is 1.0 h later than that of LK638S, which may be a result of the suppression of JA biosynthesis and signaling in the *gom1* mutant.

The loss of function of genes in the JA biosynthesis pathway resulted in a variety of male-sterile mutants in *Arabidopsis* [12,39,45,46,47]. ORR3 catalyzes a reductive reaction from 12-oxophytodienoic acid (OPDA) to jasmonoyl-isoleucine (JAIle). The *opr3-3* allele is responsible for completely blocking JA biosynthesis, resulting in male sterility in the *opr3-3* mutant [46]. Knockout of the rice *OsOPR7* gene, an ortholog of *Arabidopsis OPR3* essential for JA biosynthesis, led to male sterility that was restored by exogenous MeJA application [4]. In this study, the seed setting rate of the *gom1* mutant is only 15.6%, while that of the wild-type LK638S is 79.5% (Figure S1). These results suggested that the fertility of the *gom1* mutant was impaired, which may be due to the inhibition of JA biosynthesis and signaling in the *gom1* mutant.

In summary, we present the role of GOM1 in regulating rice glume-opening and closing. The *gom1* mutant maintains a high osmotic pressure caused by carbohydrate accumulation through JA biosynthesis and signaling after anthesis, leading to the lodicules swelling and glume-opening.

## 4. Materials and Methods

### 4.1. Plant Materials and Growth Conditions

The *gom1* mutant was obtained from a ^60^Co-irradiated M_2_ population of TGMS Longke 638S (LK638S) according to the method described by Adewusi et al. (2021) [48]. The F_2_ mapping population was derived from a cross between *gom1* and Guangzhan63-4S. Guangzhan63-4S is an elite TGMS line that is widely used in two-line hybrid rice. TGMS lines are male-sterile under high-temperature conditions but convert to male-fertile under low-temperature conditions. Thus, TGMS can self-pollinate plants under low-temperature conditions. At the heading stage, F_2_ plants with the *gom1* phenotype were used for gene mapping. Rice (*Oryza sativa*) plants were grown in the experimental field during the natural growing season at Changsha or Lingshui. Transgenic plants were grown in pots in a greenhouse under standard growth conditions.

### 4.2. Map-Based Cloning of GOM1

The *GOM1* locus was mapped using 1361 mutant F_2_ plants selected from the progeny of a cross between *gom1* and Guangzhan63-4S. Genomic DNA was extracted from the leaves of F_2_ plants, and polymerase chain reaction (PCR) was performed with 2× Taq Master Mix (Novoprotein, Suzhou, China) under the following conditions: 20 s denaturation at 94 °C, 20 s annealing at 60 °C, and 30 s extension at 72 °C, 30 cycles. We mapped the target locus by a combination of over 120 polymorphic SSR markers evenly distributed over the whole genome and InDel markers designed by ourselves. Primers used for generating these markers are listed in Supplemental Table S1. Second-generation genome resequencing of LK638S and the *gom1* mutant was performed, and their sequences were compared in candidate intervals to identify SNP and InDel mutation sites (Table S2).

### 4.3. Vector Construction and Transgenic Analysis

For the genetic complementation test, a 7.3-kb genomic fragment covering the entire coding region of *GOM1*, plus a 2589 bp upstream sequence and 314 bp downstream sequence, was amplified by PCR from LK638S with the primer pair GOM1-infusion-F and GOM1-infusion-R, and inserted into the binary vector pCAMBIA1390 using the In-Fusion Cloning method (Takara, Beijing, China). To generate the *GOM1* CRISPR/Cas9 construct, a 20-bp sgRNA targeting the six exons of GOM1 was cloned into the CRISPR/Cas9 expression vector according to a method previously described [5]. The resulting constructs were introduced into LK638S by *Agrobacterium tumefaciens*-mediated transformation, as described previously [49]. The primers used to create the above constructs are listed in Table S1. The CRISPR/Cas9 transgenic plants were genotyped by Sanger sequencing of the PCR product amplified with primers GOM1-CX-2F and GOM1-CX-2R.

### 4.4. Promoter-GUS Analysis

For promoter activity analysis, a 2000-bp genomic fragment upstream of the ATG start codon was PCR-amplified using Phanta Max Super-Fidelity DNA Polymerase (Vazyme, Nanjing, China) from kitaake genomic DNA with the primer pair ProGUS-GOM1-F and ProGUS-GOM1-R (Table S1) and fused to the GUS reporter gene in the binary vector pCAMBIA1301. The resulting construct p*GOM1*::GUS was introduced into the kitaake by the *Agrobacterium tumefaciens*-mediated transformation method [49]. Histochemical staining of GUS activity in transgenic plants was performed as previously described [50].

### 4.5. JA Measurement

Lodicules were detached from spikelets of LK638S and *gom1* mutant and immediately frozen in liquid nitrogen for use in endogenous JA measurements. The content of JA was determined by Wuhan Greensword Creation Technology Co., Ltd. (Wuhan, China) (http://www.greenswordcreation.com, accessed on 29 September 2024) based on UHPLC-MS/MS analysis (Thermo Scientific Ultimate 3000 UHPLC coupled with TSQ Quantiva).

### 4.6. Determination of Soluble Sugar Contents and Osmolality

Lodicules of LK638S and *gom1* mutant at different stages of anthesis were harvested from spikelets and thoroughly ground fully in liquid nitrogen. After lyophilization, about 100 mg of ground material was used for the measurement of sugar content based on previously published methods [51,52], and the osmolarity of lodicules was also measured using an FM-9J osmometer according to the manufacturer’s instructions (Yida, Shanghai, China).

### 4.7. RNA Sequencing (RNA-Seq) Analysis

For RNA-seq analysis, lodicules of LK638S and *gom1* mutant were collected at the AA2 stage, and total RNA extraction was carried out. Library preparation and Illumina sequencing were conducted by Novogene Co., Ltd. (Beijing, China), using three biological replicates of each sample. Raw reads were trimmed using cutadapt to obtain clean reads [53]. Subsequently, the trimmed reads were aligned to the reference rice genome obtained from RAP-DB (https://rapdb.dna.affrc.go.jp, accessed on 29 September 2024). Differentially expressed genes (DEGs) were identified based on a ≥two-fold expression change and a *p*-value < 0.05. Genes that exhibited downregulation or upregulation between LK638S and *gom1* mutants were further subjected to gene ontology (GO) enrichment analysis using ClusterProfiler [54]. The expression levels of the selected DEGs were validated by RT-qPCR.

### 4.8. RNA Isolation and RT-qPCR

Total RNA was isolated using TRIzol reagent (Sangon Biotech, Shanghai, China), with subsequent treatment using gDNA Eraser to remove any DNA contamination. The quality of total RNA was assessed using a NanoDrop 2000 spectrophotometer (Thermo Fisher Scientific, Shanghai, China). First-strand cDNAs were synthesized using 1 μg of total RNA as a template, according to the manufacturer’s instructions for the PrimeScript RT reagent kit (Takara, Beijing, China). RT-qPCR was conducted on an ABI 7500 Real-Time PCR System (Applied Biosystems, Foster City, USA) using TB Green Premix Ex Taq II (Takara, Beijing, China) to monitor dsDNA synthesis, according to the manufacturer’s instructions. The primer sequences used for RT-qPCR are listed in Table S1. The expression levels of the rice *Actin* gene (LOC_Os03g50885) served as internal controls. Relative expression levels were determined as previously described [55].

### 4.9. Dynamic Observation and Analysis of Lodicule Cell Size

The florets of LK638S and *gom1* mutant were collected at various time periods of the day. A pair of lodicules was carefully peeled from the lemma and observed and photographed using a dissecting microscope (Olympus SZX10, Tokyo, Japan). The peeled lodicules were subsequently placed on a slide with the front side facing upwards. Photographs of the cells were taken using a microscope (Echo, San Diego, CA, USA), the number of cells was counted, and the average cell area was calculated.

## 5. Conclusions

Opened glume during anthesis is a serious problem in the seed production of rice. In the present study, we identified a rice *gom1* mutant that displayed glume-opening with unwithered lodicules post-anthesis. Molecular cloning revealed that the *gom1* mutant harbors a mutation in GOM1, which encodes a receptor-like kinase (RLK) expressed in the lemma, palea, lodicule, pistil, and stamen. The diurnal flower opening time (DFOT) of the *gom1* mutant is 1.0 h later than that of LK638S. Loss-of-function of *GOM1* resulted in a reduction in the gene expression related to JA biosynthesis, JA signaling, and sugar transport. Compared with LK638S, the JA content in the *gom1* mutant was significantly reduced, while the carbohydrate content was significantly increased in lodicules after anthesis. In conclusion, we speculated that GOM1 regulated carbohydrate transport in lodicules during anthesis through JA biosynthesis and signaling, maintaining a higher osmolality in lodicules, leading to glum-opening after anthesis.

## Data Availability

The original contributions presented in the study are included in the article/supplementary material, further inquiries can be directed to the corresponding author.

## Supplementary Materials

The following supporting information can be downloaded at https://www.mdpi.com/article/10.3390/plants14010005/s1, Figure S1. Comparison of major agronomic traits between LK638S and gom1 mutant in Lingshui. Figure S2. CRISPR/Cas9-induced mutations in the GOM1 genes. Figure S3. Alignment of the amino acid sequence of GOM1 between LK638S and mutant lines. Table S1. List of primers used in this study. Table S2. Variation information of candidate intervals. Table S3. Differential expression data of genes related to JA synthesis, JA signaling, and sugar transport in RNA-seq.
