# Supplementary material for: Receptor-like Kinase GOM1 Regulates Glume-Opening in Rice
Source: Plants (Basel). 2024 Dec 24;14(1):5. doi: 10.3390/plants14010005 (PMC11722787; doi:10.3390/plants14010005)
Supplement: Supplementary file 1 [file plants-14-00005-s001.zip › plants-3371098-supplementary.pdf]

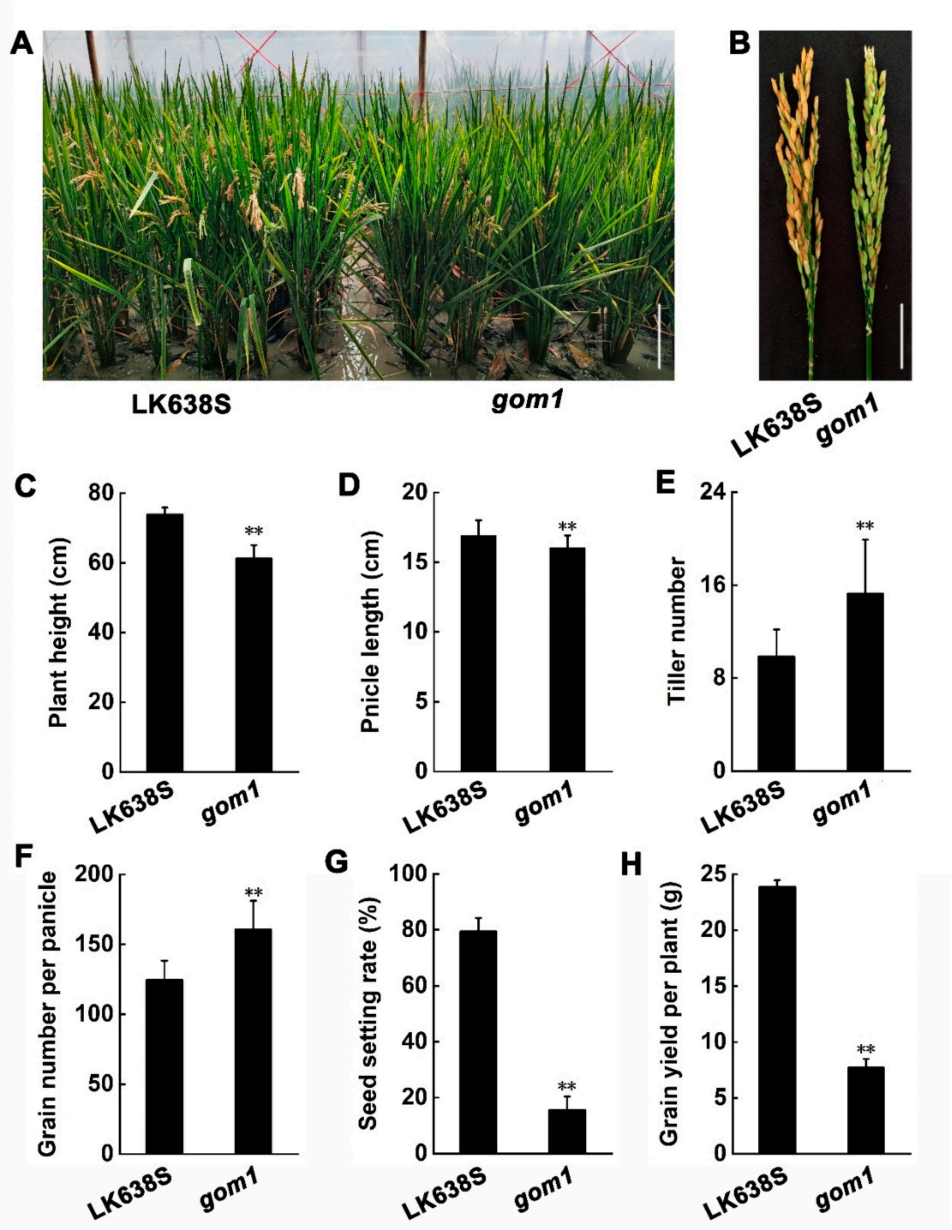

Figure S1. Comparison of major agronomic traits between LK638S and *gom1* mutant in Lingshui. (A) Phenotypes of LK638S and *gom1* mutant plant at the heading stage. Scale bar, 20 cm. (C-H) The major traits including plant height (C), panicle length (D), tiller number (E), grain number per panicle (F), seed setting rate (G) and grain yield per plant (H) are showed in histograms. Agronomic traits were investigated in paddy filed located at Poliu village (18°30′44″N, 110°25′39″E), Lingshui, in 2023. Data are presented as mean ± SD (n = 20). ** indicates a significant difference (P < 0.01 from Student's *t*-test).

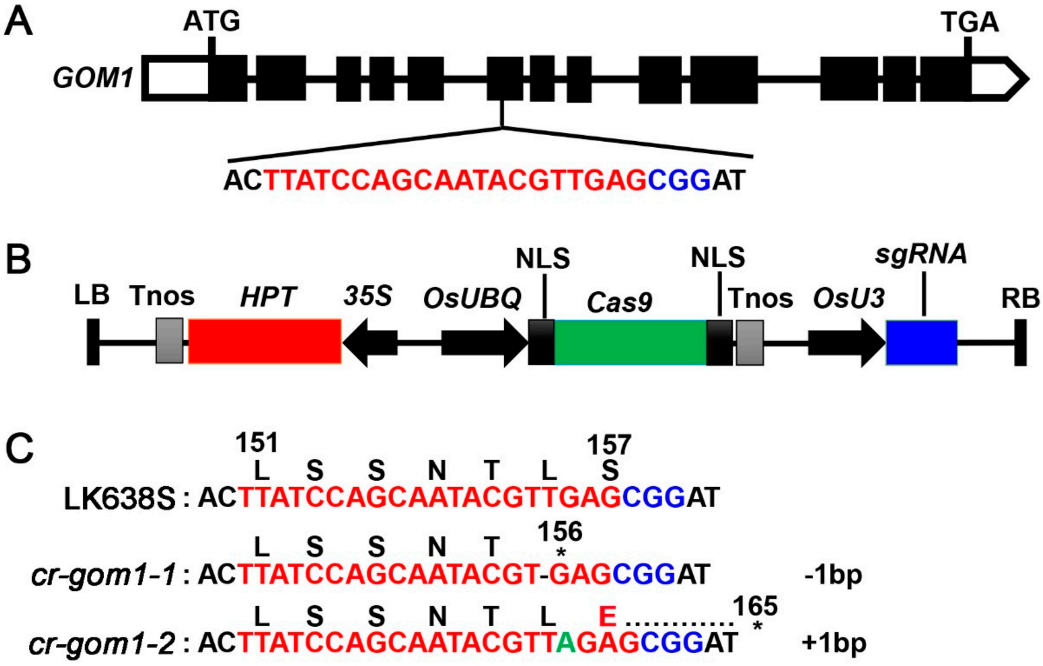

Figure S2. CRISPR/Cas9-induced mutations in the *GOM1* genes. (A) Schematic of the *GOM1* gene structures and target site. Exons and introns are indicated with black rectangles and black lines, respectively. The spacer and PAM sequences were marked in red and blue. (B) Schematic diagram of the T-DNA structures including the Cas9 and single sgRNA in genome editing construct. The expression of Cas9 is driven by the maize ubiquitin promoter (*OsUBQ*); the expression of the sgRNA scaffold is driven by the rice *OsU3* small nuclear RNA promoter; the expression of hygromycin (*HPT*) is driven by CaMV35S promoter. NLS, nuclear localization signal; Tnos, the terminator; LB and RB, left border and right border, respectively. (C) Homozygous mutations identified at the target sites of *gom1* mutant lines in the T_1 generation. Amino acids were marked above the relative nucleotide triplets, and the first altered ones from the frameshift were indicated in red, with the number representing the order in the proteins. The stars represent premature stop codons.

```
  LK638S MGSFLRKQPSFLLILLILHLGAREARALSSDGEALLAFKKAVTTSDGIFLNWREQDVDPCNWKGVGCDSHTKRVVCLILAYHKLVGPIPP   90
cr-gom1-1 MGSFLRKQPSFLLILLILHLGAREARALSSDGEALLAFKKAVTTSDGIFLNWREQDVDPCNWKGVGCDSHTKRVVCLILAYHKLVGPIPP   90
cr-gom1-2 MGSFLRKQPSFLLILLILHLGAREARALSSDGEALLAFKKAVTTSDGIFLNWREQDVDPCNWKGVGCDSHTKRVVCLILAYHKLVGPIPP   90

  LK638S EIGRLNQLQALSLQGNSLYGSLPPELGNCTKLQQLYLQGNYLSGHIPSEFGDLVELGTLDLSSNTLSGSIPPSLDKLAKLTSFNVSMNFL  180
cr-gom1-1 EIGRLNQLQALSLQGNSLYGSLPPELGNCTKLQQLYLQGNYLSGHIPSEFGDLVELGTLDLSSNT.........................  155
cr-gom1-2 EIGRLNQLQALSLQGNSLYGSLPPELGNCTKLQQLYLQGNYLSGHIPSEFGDLVELGTLDLSSNTLER.IDSTLS...............  164

  LK638S TGAIPSDGSLVNFNETSFIGNRGLCGKQINSVCKDALQSPSNGPLPPSADDFINRRNGKNSTRLVISAVATVGALLLVALMCFWGCFLYK  270
cr-gom1-1 ..........................................................................................  155
cr-gom1-2 ..........................................................................................  164

  LK638S NFGKKDIHGFRVELCGGSSIVMFHGDLPYSTKEILKKLETMDDENIIGVGGFGTVYKLAMDDGNVFALKRIMKTNEGLGQFFDRELEILG  360
cr-gom1-1 ..........................................................................................  155
cr-gom1-2 ..........................................................................................  164

  LK638S SVKHRYLVNLRGYCNSPSSKLLIYDYLPGGNLDEVLHEKSEQLDWDARINIILGAAKGLAYLHHDCSPRIIHRDIKSSNILLDGNFEARV  450
cr-gom1-1 ..........................................................................................  155
cr-gom1-2 ..........................................................................................  164

  LK638S SDFGLAKLLEDDKSHITTIVAGTFGYLAPEYMQSGRATEKTDVYSFGVLLLEILSGKRPTDASFIEKGLNIVGWLNFLVGENREREIVDP  540
cr-gom1-1 ..........................................................................................  155
cr-gom1-2 ..........................................................................................  164

  LK638S YCEGVQIETLDALLSLAKQCVSSLPEERPTMHRVVQMLESDVITPCPSDFYDSE                                      594
cr-gom1-1 ......................................................                                      155
cr-gom1-2 ......................................................                                      164
```

Figure S3. Alignment of the amino acid sequence of GOM1 between LK638S and mutant lines. The dark blue and light blue shading indicate 100% and ≥50% similarity, respectively.

Table S1. List of primers used in this study

Primer	Sequence (5'–3')
RM14616-F	CCGAATCTGTGTTCTCCAATTCC
RM14616-R	CAACCAAAGAGAACCAACCAAGC
RM14795-F	GCGCGATAAACGTTTGAGAGAAGG
RM14795-R	AAATCAGTCGCGGTTGCTGTCC
RM14686-F	AGGAGGAAGGAAGAACAGAGTTGC
RM14686-R	CTGAGTGCGTGCCATTTATTTCC
RM14737-F	CAGCTGCAGGAGAGAAGACAGG
RM14737-R	CTCTCCAAACATTCTTCCCAACC
RM14761-F	GAGCGCCCGATTTGTAGATGC
RM14761-R	ATATCCTTTCCACCTGACATCCTTGC
L3-19-F	CCGTATGCTTTAATTTAGCCCTTCT
L3-19-R	CAATAATACGGGTGACACGTAAAT
S3-9-F	GGGCGATCAAGTTTCTGG
S3-9-R	TCGATGTTCCTGCCGTTC
S3-15-F	GTGTTCGCCTGCTCGTCA
S3-15-R	AAAGTCCTTGCTGGTGGG
GOM1-infusion-F	CCGGCGCGCCAAGCTTAGGATGGGTCTTCGGTGCGT
GOM1-infusion-R	GAATTCCCGGGGATCCTCTAGCAGCAGCTCATGGAG
U3-GOM1-F	GGCATTATCCAGCAATACGTTGAG
U3-GOM1-R	AAACCTCAACGTATTGCTGGATAA
GOM1-CX-2F	ATCAGACTGATCTCCCCTGTCTG
GOM1-CX-2R	GGTTCATGAAAGGAACTGAAAAGA
ProGUS-GOM1-F	CCCAAGCTTAACGGGAGCAGTGGTGTGG
ProGUS-GOM1-R	CATGCCATGGTTTTCACCAGTTGGTGAAGCATATA
GOM1-qPCR-F	GTTGCAGGAACATTTGGCTATC
GOM1-qPCR-R	CCCGATTCTCACCGACCAG
OsAOC-qPCR-F	CCAGGTCAAGCTCAACCAGAT
OsAOC-qPCR-R	GTCGGGGATGCCCTTGAG

OsOPR1-qPCR-F	GTCTCCACCACAGATTTCCAGC
OsOPR1-qPCR-R	AAGTCGTCGATGATCTGAGGGA
OsOPR7-qPCR-F	GAAGAAGGTGGTGGATGCTGT
OsOPR7-qPCR-R	GGTTTAGGATACTTGCCATAGGAG
OsJAZ1-qPCR-F	TCTGTAGCAACTGGACAACCTCA
OsJAZ1-qPCR-R	TGCTGTCACGGCGTTTCTC
OsJAZ6-qPCR-F	AACCTGTTCGTCCAAATGCTG
OsJAZ6-qPCR-R	TGAAGGCGATCCTTTCTTTTCT
OsJAZ11-qPCR-F	AAGGAGCACAGTGGAAGCAAC
OsJAZ11-qPCR-R	TGCGTGTCTTTCAGCGTCC
OsJAZ12-qPCR-F	GAAGAGGAAGCACAGGATCACGA
OsJAZ12-qPCR-R	CCTTCTTGTATGGTTCGCTCGTT
OsJAZ13-qPCR-F	AGACGAGCGGGAGGAGGAA
OsJAZ13-qPCR-R	CCCTTCCTCTTCTCCATGAACC
SWEET4-qPCR-F	AAGGGGTCGGTGGAGCAGT
SWEET4-qPCR-R	AAGAGGGCGATGTAGGTGAGC
SWEET11-qPCR-F	CGACTCCATGTCCCCGATCT
SWEET11-qPCR-R	CACGGACAGGATGGTGAAGG
SWEET15-qPCR-F	TGGATGACGAGGAGACGAACC
SWEET15-qPCR-R	CATGTCAGGCTTGATGATCTGCT
SWEET16-qPCR-F	GAGCAAGAGCACGGAGGAAT
SWEET16-qPCR-R	CGAGGTAGAGCGTGACGTAGATG
OsACTIN-qPCR-F	ACCCAAGAATGCTAAGCCAAGAG
OsACTIN-qPCR-R	ACTTTGTCCACGCTAATGAAGAAAC

Table S2. Variation information of candidate intervals

Chr	Position	LK638S	*gom1*	Gene ID	Variation type
3	8799845	G	GCT	LOC_Os03g15940	Downstream_gene_variant
3	8824857	T	G	LOC_Os03g16000	Downstream_gene_variant
3	8830228	TC	T	LOC_Os03g16010	Frameshift_variant

Table S3. Differential expression data of genes related to JA synthesis, JA signaling, and sugar transport in RNA-seq

Gene ID	Gene Name	LK638S readcount	*gom1* readcount	Up/Down-Regulation (*gom1*/LK638S)	P-value	log2FoldChange (*gom1*/LK638S)
LOC_Os03g32314	*OsAOC*	11634.58	3804.63	Down	2.74E-14	-1.6123
LOC_Os06g11290	*OsOPR1*	24.01	5.42	Down	0.031568	-2.1626
LOC_Os08g35740	*OsOPR7*	6920.40	3455.31	Down	2.24E-09	-1.0018
LOC_Os04g55920	*OsJAZ1*	7638.84	3158.21	Down	2.75E-18	-1.2738
LOC_Os03g28940	*OsJAZ6*	9820.01	3124.90	Down	5.74E-17	-1.6516
LOC_Os03g08320	*OsJAZ11*	16764.67	4574.03	Down	6.07E-16	-1.8738
LOC_Os10g25290	*OsJAZ12*	4808.17	1674.25	Down	4.29E-10	-1.5218
LOC_Os10g25230	*OsJAZ13*	1531.01	229.97	Down	5.25E-06	-2.7343
LOC_Os02g19820	*OsSWEET4*	120975.26	48603.70	Down	3.43E-29	-1.3155
LOC_Os08g42350	*OsSWEET11*	14037.27	5926.75	Down	0.019232	-1.2352
LOC_Os02g30910	*OsSWEET15*	8113.66	3744.92	Down	1.03E-10	-1.1157
LOC_Os03g22200	*OsSWEET16*	7589.74	3675.60	Down	2.44E-05	-1.046
